# Change in Acoustic Parameters of Electric Guitar Strings Under Dynamic Loading

**DOI:** 10.3390/s25133989

**Published:** 2025-06-26

**Authors:** Jakub Grzybowski, Piotr Wrzeciono, Hydayatullah Bayat

**Affiliations:** 1 Faculty of Applied Informatics and Mathematics, Warsaw University of Life Sciences, 02-776 Warsaw, Poland; 2 Institute of Information Technology, Warsaw University of Life Sciences, 02-776 Warsaw, Poland; piotr_wrzeciono@sggw.edu.pl; 3 Institute of Civil Engineering, Warsaw University of Life Sciences, 02-776 Warsaw, Poland; hydayatullah_bayat@sggw.edu.pl

**Keywords:** guitar, string, dynamic strength, static strength, material deterioration

## Abstract

The aim of our work was to investigate how electric guitar strings wear out. There are many myths about string wear. We decided to investigate what the wear process looks like in real life. In our work, sound processing methods such as DTFT and spectrogram were used. However, the most important research method is the use of time-frequency analysis to study the sound of the string and its wear process. Another key method used in our work is the application of a phenomenon known from psychoacoustics, pitch. In our work, we have been able to show that the use of pitch in combination with time-frequency analysis makes it possible to demonstrate string wear. This was not achievable for previously known methods. We have also shown that the string yield limit is exceeded immediately when the strings are placed on the guitar neck. This affects the sound equation of the string. In this work, we have proposed a transformation of the classical string equation so that it correctly describes the sound of the string as it is worn. The research method we have developed, combining pitch and time-frequency analysis, could presumably be used in the future to study the wear and tear of other vibrating systems, such as bridges and viaducts.

## 1. Introduction

### 1.1. Electric Guitar History

The electric guitar is one of the most popular musical instruments. Electric guitars have shaped modern music since their emergence in the early 20th century. Unlike acoustic guitars, which rely on a hollow body to amplify sound, electric guitars use electromagnetic pickups to convert string vibrations into electrical signals, which are then amplified through external devices. This technological innovation has allowed for greater control over volume and tone, paving the way for the development of diverse musical genres [1].

The first commercially successful electric guitar was the Rickenbacker “Frying Pan” (1931), followed by significant models such as the Gibson ES-150 and Fender Telecaster. Leo Fender’s introduction of the solid-body Fender Stratocaster in 1954 marked a turning point in guitar design, offering players a sleek, durable instrument with versatile tonal capabilities [2]. These innovations facilitated the rise of rock and roll, blues, jazz, and later, heavy metal, and progressive music.

A typical electric guitar features a solid body, magnetic pickups, volume and tone control knobs, and a bridge system that may include tremolo mechanisms. Various pickup configurations (single-coil, humbucker) allow for a broad spectrum of sounds, from bright and sharp to warm and distorted.

The influence of electric guitars extends beyond music into cultural and social realms. Iconic artists such as Jimi Hendrix, Eric Clapton, and Jimmy Page helped define entire eras with their distinctive guitar styles and sound innovations [3].

Today, electric guitars remain at the forefront of musical expression, with modern advancements incorporating digital effects, MIDI capability, and ergonomic design improvements. They continue to inspire musicians across genres and generations [4].

### 1.2. Aim of Research

The electric guitar can be divided into two parts in a very general way. The strings, which vibrate, and the rest of the instrument. There are many papers related to the body of the instrument, for example [5,6,7,8,9,10]. On the other hand, few papers have been written on electric guitar strings, and those that exist do not deal with issues that interest musicians.

A popular claim among musicians is that an electric guitar’s strings last about 40 h of use [11]. After this time, their properties change so that the quality of the guitar’s sound is affected, and it is necessary to consider changing them.

We decided to test this hypothesis. We started by analyzing the physical phenomena that can affect string wear. Tension, vibration, and pressure of the string against the fret affect the distribution of the string’s linear density and thus the sound it produces. Changing the distribution of the string’s linear density causes the string to stop tuning correctly along the length of the guitar neck. In addition, the sound quality they produce deteriorates with wear.

To study how the sound produced by the string and the string itself change, we planned three experiments to show the wear and tear of strings with use. The first of the experiments was to test the yield strength of the string we tested. This test showed us how the force of tension affects the stretching of the string. The following experiment was to study the changes in the thickness of a new and worn string. The thickness of the worn string could vary by up to a few processes relative to the thickness of the new string, which was similar along the entire string length. The last of our experiments was a study of the sound produced by electric guitar strings. The sounds of the strings were recorded after each hour of playing the guitar until the end of the experiment.

Then we applied the discrete-time Fourier transform (DTFT) [12]. The resulting recordings were used to determine the pitch using an appropriate perception model. The determined pitch allowed us to observe how the sound produced by the string behaves over time.

## 2. Guitar Construction and Playing Techniques

**Figure 1:**
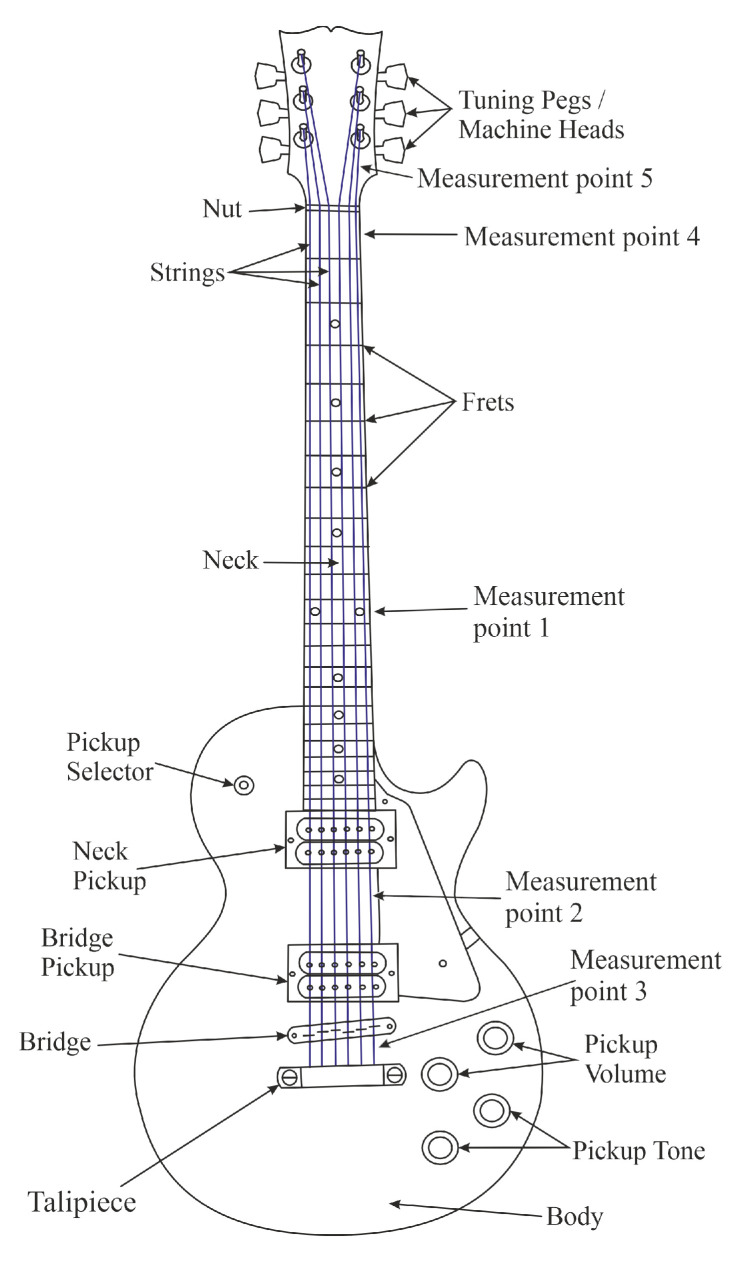
Diagram of guitar electric guitar construction.

In order to better understand the rest of the article, one must familiarize oneself with the guitar structure shown in Figure 1 [13]. From the paper’s point of view, the significant parts of the guitar are: strings, tuning machines, guitar nut, bridge, and tailpiece.

The article focuses on the study of strings. For the sound quality to be as high and repeatable as possible, a high-quality instrument is necessary [14]. In our experiments, we used a guitar from one of the top guitar manufacturers.

### 2.1. Structure and Types of Strings

The electric guitar has six strings, which are usually tuned E2, A, D, G, B, and E4 (*f* = 82.41, 110.00, 146.83, 196.00, 246.94, 329.63 Hz). We will use this terminology in the rest of the paper.

Strings used in experiments can be divided into two types. The first type of string is a wrapped string. Wrapped strings include E2, A, and D strings. Sometimes, the G string is also wrapped. The second type of strings is strings without a wrapper. Strings without a wrapper include G, B, and E4 strings.

#### 2.1.1. Wrapped Strings

**Figure 2:**
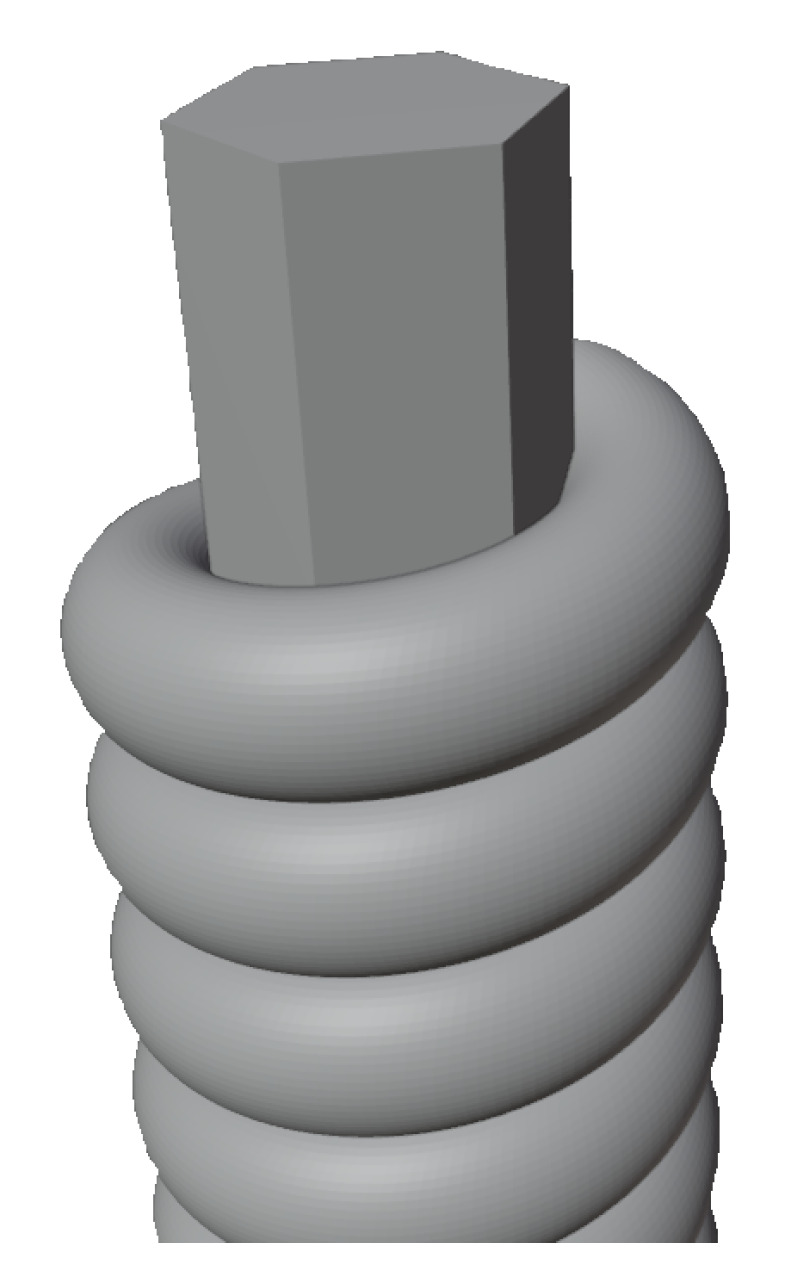
Scheme of string construction with wrapper.

The wrapped strings used in the experiment are constructed of two elements. Wrapped strings have a hexagonal core made of carbon steel alloy. A wrapper made of nickel-plated steel wire is wound onto this core. The construction of a wrapped string is shown in Figure 2. The structure of wrapped strings and the chemical composition of wrapped strings have been studied and described in the paper [15].

#### 2.1.2. Strings Without Wrapping

The unwrapped strings used in the experiment have a cylindrical carbon steel core. This core is additionally coated with a thin layer of nickel. The construction of the unwrapped strings and the chemical composition of the unwrapped strings were studied and described in the paper [15].

### 2.2. Playing Techniques

There are many techniques for playing the guitar. Since we use an electric guitar, we must distinguish several playing techniques, such as alternate picking, palm muting, pull-up, down picking, or tapping.

Alternate picking involves plucking the strings alternately from above and below. Palm muting involves muffling the sound of a strum with the right hand resting on the guitar’s bridge. Pulling up involves pushing the resonating string up (or sometimes down) with the fingers of the left hand to make the sound half a tone higher than before pulling up. Downpicking involves strumming the strings with the guitar pick only from top to bottom. Tapping involves strumming the strings to sound using the fingers of the right hand on the guitar’s fret.

## 3. Experiments

In all experiments, we used two sets of strings available from a popular manufacturer. We called them sets A and B. This nomenclature will be used in the rest of the article. Set A of strings came from the mid-price range for electric guitar strings, and Set B came from the much higher price range for electric guitar strings.

We decided to conduct three experiments. The first experiment studied the string’s recorded sound and wear. The second measured the change in string thickness. The third studied the string’s yield stress. The first two experiments were conducted on two sets of strings from a popular manufacturer’s range. The yield strength of the strings was tested for the A set.

### 3.1. The Way Strings Are Used

The study aimed to investigate the wear of strings operated utilitarianly. However, some rules were introduced for the playing sessions to maintain an even wear rate of the strings. We have described these rules in detail in Appendix A.

Each session consisted of playing the guitar for one hour. The sound of the whole string of each string was recorded. We also recorded chromatic scales on each string separately.

An hour of playing was divided into three parts, 20 min each. The first 20 min were devoted to playing pentatonic scales [16]. A pentatonic scale is a scale made up of 5 notes within an octave. The central sound of a pentatonic scale is the middle sound, supplemented by intervals of a major second and a diminished fourth, both up and down. The pentatonic scales engaged all the strings of the guitar equally. Another 20 min were devoted to playing riffs [17]. A riff in music refers to a short melody, motif, or single phrase repeated many times within a song. The riffs mainly involved the E2, A, and D strings. The last 20 min of the playing were devoted to playing guitar solos [18]. The solos mainly engaged the G, H, and E4 strings.

Thanks to the introduced rules, the tested strings were exploited in a controlled and still usable manner. Collecting data after each hour of exercise allowed us to observe the strings’ wear process during their exploitation.

### 3.2. Duration of the Experiment

While observing the behavior of the strings, we decided that the experiment would last 30 h for each set of strings. This decision was made based on expert opinion and the tuner’s observation while tuning the guitar strings. While tuning the worn string, the tuner showed significant changes in the sound frequency produced for the first few seconds. A set of strings was no longer playable after 30 h of use.

### 3.3. Recording Audio Data After Each Recording Session

The new strings lay rolled up in their packaging. For the strings to obtain the right properties, they need to stretch and sit properly on the bridge and saddle of the guitar. So it was decided that the first recording of the strings’ sounds would be made after using them for 2 h. This time allows the strings to align correctly and achieve the desired characteristics. According to what was written in Section 3.1, the last recording was after 30 h of using the strings.

A recording session followed each hour of guitar playing. The tone of each whole string and chromatic scales were recorded during the recording session. These recordings were made twice during each session. We record sounds twice to minimize the influence of possible external factors, such as inappropriate string pressing by the musician. The data were collected using a Behringer UMC204HD. Parameters of the sound files(fs = 44.1 kHz, number of bits = 32).

### 3.4. Changing the Tune Quality

While experimenting, we noticed freshly tuned strings and their wear stopped tuning along the entire neck length. The string was perfectly tuned for its whole string and stopped tuning at the tested 12th fret. A string after 2 h of exploitation and after tuning, tunes perfectly on all frets. On the other hand, after 30 h of playing, tuned perfectly on its whole string, stops tuning on the 12th fret. This phenomenon was observed even on a guitar tuner. The described effect was observed after 16 h of string use for a set of A strings. For set B, on the other hand, the phenomenon was considered only after 21 h of operation. The first string on which the phenomenon occurred was the E2 string. Subsequently, the phenomenon began to occur on the other strings.

### 3.5. Difficulty in Tuning Worn Strings

Newly put on and placed on the neck strings (after two hours of playing), we could tune easily; tuning the strings took a few seconds at most. As the time of string use increased, it took us longer to tune the strings. The longer tuning time was due to the greater fluctuation of the frequencies read by the tuner. After some time of exploitation, the wear of the string reached such a level that it was impossible to tune it. The tuner showed significant frequency fluctuations after striking the string with a pick. In order to tune such a string, it was necessary to wait a few seconds for the fluctuations to stabilize. For set A, this phenomenon was observed after 21 h of use. In contrast, it was 28 h of exploitation for a B set.

### 3.6. Change in String Thickness

After 30 h of string exploitation, the change in thickness was examined. The thickness of the strings was tested using a micrometer. Each measurement was repeated three times and taken at several locations:

1. 12th fret,
2. Picking place,
3. Behind the bridge,
4. 1st fret,
5. By the key.

The locations of the measurements are accurately shown in Figure 1.

### 3.7. Results of String Thickness Measurements

Each measurement was taken three times, and then the average was calculated. We analyzed the obtained results.

The new strings had the same dimension along their entire length, close to that stated by the manufacturer. According to the measurements taken, the strings stretched irregularly. The difference in the thickness of the strings could be as much as 7%. We can see this in Table 1 for measurements taken at the picking place. Strings having a wrap were found to be particularly susceptible to thickness variation. We considered this was due to the wrapper coming loose due to the string core deformation.

## 4. Acoustic Parameters

### 4.1. Fundamental Frequency

We initially assumed that only its fundamental frequency $f_{1}$ would suffice to classify a string’s sound, but the fundamental frequency is stable and does not change over time.

**Figure 3:**
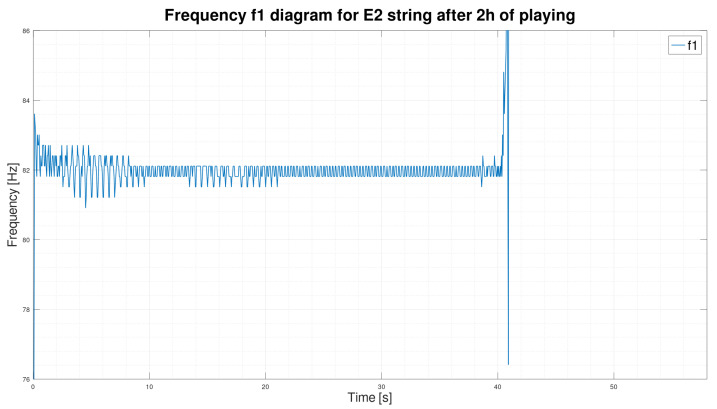
Frequency $f_{1}$ graph of the E2 string sound after 2 h of playing.

**Figure 4:**
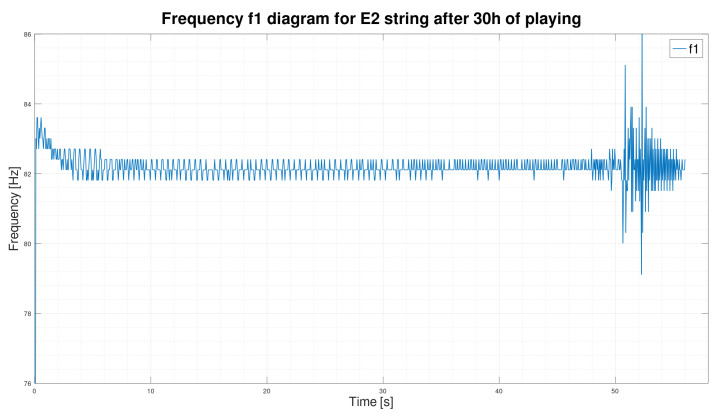
Frequency $f_{1}$ graph of the E2 string sound after 30 h of playing.

As we can see in Figure 3 and Figure 4, the frequency waveform $f_{1}$ over time is very stable. The new string, after two hours of use, rearranged in Figure 3, is not significantly different from the frequency waveform of the exact string after 30 h of playing (Figure 4). However, according to our observation of the E2 string, the string after 30 h of playing is unquestionably unsuitable for further playing. We concluded that the fundamental frequency $f_{1}$ alone is insufficient to determine the string’s wear correctly. In order to search for better solutions, we decided to determine the sound spectrograms of the string.

We used the DTFT transform to determine the fundamental frequency. The DTFT is a transform often used to analyze samples of a continuous function. The DTFT is described by the Formula (1) [12].

(1) $$X_{2\pi }\left(\omega \right)=\sum_{n=-\infty }^{\infty }x\left[n\right]e^{-j\omega n},$$

where ω=2πf—pulsation; *n*—discrete time; x[n]—sampled form of the signal x[t].

Parameters (N = 4096, fs = 44,100, window = Hamming).

### 4.2. Sound Spectrogram

A spectrogram [19] is a graph showing the amplitude spectrum of a signal over time. It is a time-frequency analysis. So, by analyzing the spectrogram, we can observe several important phenomena.

**Figure 5:**
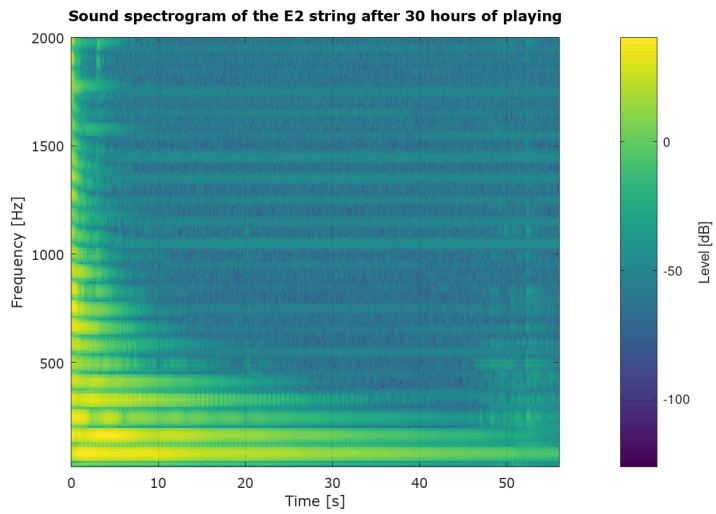
E2 string sound spectrogram after 30h of playing.

Figure 5 shows an example of a string sound spectrogram. It is the sound spectrogram of the E2 string after 30 h of exploitation. It will serve as an example to visualize the conclusions presented below.

The most important phenomena visible on the spectrogram are the changes in individual harmonics. The frequency changes of the first harmonic ($f_{1}$) is very stable. However, changes in the frequency can be seen very well in the higher harmonics. These changes are visible at the third and higher harmonics in the spectrogram (Figure 5). The third harmonic of the E2 string sound is visible at about 246Hz. For this reason, it was decided to count the pitch of the string sound rather than examine only the harmonics themselves.

Parameters (N = 4096, fs = 44,100, window = Hamming).

In the spectrogram (Figure 5), harmonics’ decay times differ. The higher the harmonic, the shorter the sounding time. We calculate the pitch using the fundamental frequency and the next three harmonics. The fundamental frequency for the E2 string can be seen on the spectrogram (Figure 5) for a frequency of 82 Hz. In order to correctly calculate pitch, all harmonics used in the calculation must end. Thus, the first conclusion is that the time of resounding should be at the same time as the time of resounding of the fourth harmonic of the sound we are studying, since it is the one that sounds the shortest of the harmonics used to calculate pitch.

In addition, the harmonics converge to their correct values for the first second of sound decay. It is particularly noticeable for the higher harmonics. The same phenomenon can be seen in the spectrogram (Figure 5), especially around 1000 Hz–1500 Hz. It was found that the impact of the guitar pick causes this. We called the stabilization time of the sound $\tau_{1}$.

With this information, we decided to use the fundamental frequency $f_{1}$ and the subsequent harmonics $f_{2}$, $f_{3}$, and $f_{4}$ for further studies.

### 4.3. Pitch

Pitch is a perceptual phenomenon related to the harmonic spectrum. For signals that do not have a harmonic spectrum, pitch can not be determined [20].

It is well known that stringed instruments are not perfectly harmonic, as shown in papers [21,22,23,24]. In paper [25], it was found that this phenomenon is due to the stiffness of the string and the pressing of the string against the fret. This phenomenon was further developed in the work [26].

The work designates the fundamental frequency as $f_{1}$. The three consecutive harmonics are designated sequentially $f_{2}$, $f_{3}$, and $f_{4}$.

To determine pitch, it is necessary to model the hearing process [20,27,28]. According to various studies [29,30], the relationships between the harmonics of a signal, not just the fundamental frequency $f_{1}$, are responsible for the impression of pitch. Based on the previously cited papers, pitch was defined as the average interval, expressed in the Formula (2).

(2) $$f\left(n\right)=\frac{\left(f_{2}\left(n\right)-f_{1}\left(n\right)\right)+\left(f_{3}\left(n\right)-f_{2}\left(n\right)\right)+\left(f_{4}\left(n\right)-f_{3}\left(n\right)\right)}{3},$$

where *f*—pitch, $f_{1}$—fundamental frequency (first harmonic), $f_{2}$—frequency of the second harmonic, $f_{3}$—frequency of the third harmonic, $f_{4}$—frequency of the fourth harmonic.

An electric guitar string has both even and odd harmonics [30,31]. Therefore, in Formula (2), it is sufficient to use the difference in frequency between the strings that are equivalent to the ideal harmonics. Due to the changes in the dimensions of the string during vibration, in the case of a real instrument, the measured harmonics differ in frequency from the ideal harmonics.

### 4.4. Time-Frequency Analysis

#### 4.4.1. The Time of Sound Resonance Calculating Method

The time of sounding will be calculated for the fourth harmonic. This approach is described in the paper [32]. We decided to take the power of the fourth harmonic’s resounding after 1 s as the initial level. The initial level was $L_{start}$. The power of the fourth harmonic was denoted $P_{f_{4}}$. The initial level was expressed by Formula (3).

(3) $$L_{start}=P_{f_{4}}\left(1\right),$$

We decided to take the initial −75 dB level as the final level. Typically, −60 dB is assumed for reverb [33], while for the electric guitar, due to relatively long bulge time and high signal-to-noise ratio (SNR), we can assume −75d B. The end level is denoted $L_{end}$. The end level is expressed by Formula (4).

(4) $$L_{end}=L_{start}-75\mathrm{dB},$$

Thus, the sounding time ends when the power of the fourth harmonic falls below $L_{end}$.

#### 4.4.2. Splitting the Sound into Three Parts

**Figure 6:**
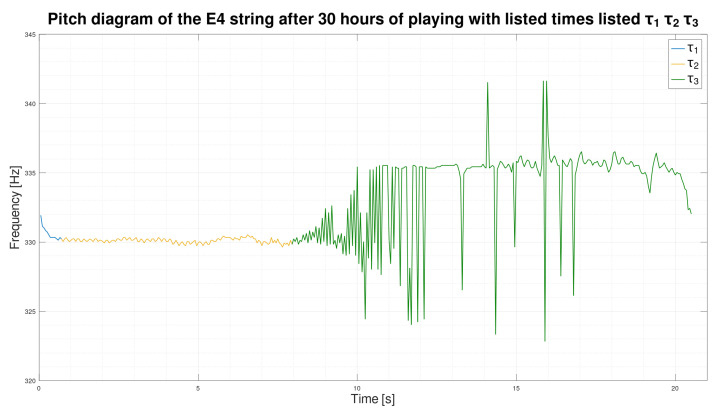
Pitch diagram with times $\tau_{1}$, $\tau_{2}$, and $\tau_{3}$.

After performing preliminary analyses, we found that dividing the time pitch into three distinct parts was possible. Figure 6 is a graph of pitch over time for an E4 string after 30 h of exploitation. Figure 6 will serve as an example to show the different parts of the pitch waveform over time.

We have divided the course of the pitch. The first part is the ankle’s impact and the sound’s stabilization after the impact. We called the sound stabilization time $\tau_{1}$. In the graph (Figure 6), $\tau_{1}$ is drawn in blue and is between 0s and 0.7s of the pitch waveform. The second part is the stable sound. We called the stable sound sounding the time $\tau_{2}$. In the graph (Figure 6), $\tau_{2}$ is drawn in yellow and falls between 0.7s and 8s of the pitch waveform. The third and at the same time the last part is the very large pitch waveform. We called the pitch modulation time $\tau_{3}$. In the graph (Figure 6), $\tau_{3}$ is drawn in green and falls between 8s and the end of the pitch waveform.

#### 4.4.3. The Sounding Times $\tau_{1}$, $\tau_{2}$ and $\tau_{3}$

It is already known that the pitch waveform can be divided into three parts, and it follows from Section 4.4.2. These parts are distinguishable on all graphs created. Therefore, it is time to give them names and define their exact ranges. So, let the part of the pitch waveform containing the hitting of the guitar pick and the stabilization of the sound after the hitting be called $\tau_{1}$. Let the fragment of the pitch waveform containing the stable sounding be named as $\tau_{2}$. We decided that the last fragment containing a pitch modulation would be called $\tau_{3}$.

#### 4.4.4. Determination of Time $\tau_{1}$

We determined the maximum value for $\tau_{1}$ in the first step. This limit was determined experimentally. After observing many graphs containing pitch waveforms of different strings. We decided that this limit should be set to 1 s. Thus, the time $\tau_{1}$ is in the range of 0 s–1 s of the pitch waveform.

#### 4.4.5. Determination of the Boundary Between Times $\tau_{2}$ and $\tau_{3}$

Then, we draw the boundaries between times $\tau_{2}$ and $\tau_{3}$. For this purpose, we calculated an estimate similar to that used in the finite difference method [34]. The estimate was calculated between the sound heights of adjacent windows. The estimate is expressed by Formula (5).

(5) $$Estimate\left(n\right)=\frac{p(n-1)-p(n+1)}{2},$$

where *p*—peach, *n*—window number.

From the observations, the results for a stable sound decay, the estimate of the difference quotient is less than 1. However, when the pitch begins to wave, the estimate of the difference quotient begins to exceed the value of 1 rapidly. Thus, we decided to set the boundary between times $\tau_{2}$ and $\tau_{3}$, when the difference quotient exceeds the value of 1 for the third time, to avoid individual anomalies inside the time $\tau_{2}$.

#### 4.4.6. End of $\tau_{3}$ Time

It was decided to choose the end of the decay time of the fourth harmonic as the termination for time $\tau_{3}$. This decision was made due to the way pitch is calculated. For the pitch to be well calculated, all harmonics must sound out. Since the fourth harmonic resounds the shortest, its resounding will be the end of the resounding of the whole sound for us.

#### 4.4.7. Comparison of the Pitch of a Worn String and a New One

We will now show how the graph and pitch parameters of a new and worn string change.

**Figure 7:**
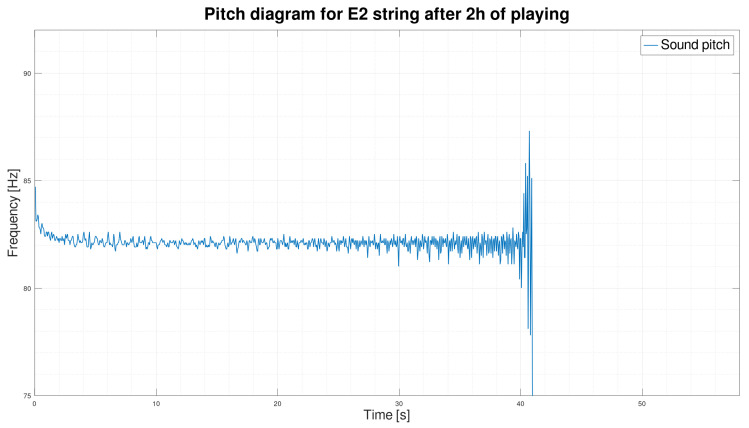
Graph of E2 string pitch after 2 h of playing.

**Figure 8:**
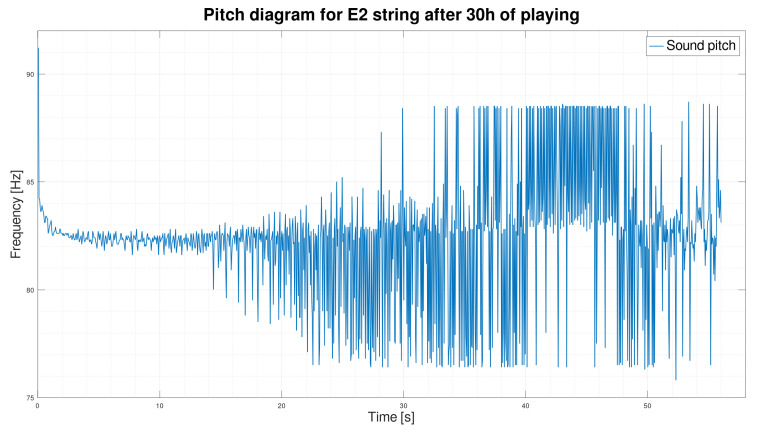
Graph of E2 string pitch after 30 h of playing.

On the diagrams, we have shown the E2 string after 2 h of playing (Figure 7) and 30 h of playing (Figure 8). We can see by analyzing the graph that there is a significant shortening of the time $\tau_{2}$ in favor of the time $\tau_{3}$. By analyzing the parameters, we can see that for string E2, after two hours of play, the standard deviations inside the times $\tau_{2}$ and $\tau_{3}$ are also smaller than those calculated for the exact string after 30 h of play.

### 4.5. Parameters and Statistical Analysis

#### 4.5.1. String Parameters

In the end, we determined six parameters that describe the string’s wear.

The first is the previously described string sounding time called $T_{f4}$. The next two parameters are the standard deviations inside $\tau_{2}$ and $\tau_{3}$ named $\sigma_{2}$ and $\sigma_{3}$, calculated by Formula (6).

(6) $$\sigma =\sqrt{\frac{\sum_{i=1}^{n}{\left(x_{i}-x\right)}^{2}}{n-1}},$$

We also calculated the average pitch inside $t_{2}$, the fourth parameter. The last two parameters that we decided to use are $\tau_{2rel}$ and $\tau_{3rel}$. They are calculated and described by Formulas (7) and (8).

(7) $$\tau_{2rel}=\frac{len(\tau_{2})}{len\left(\tau_{2}\right)+len\left(\tau_{3}\right)},$$

(8) $$\tau_{3rel}=\frac{len(\tau_{3})}{len\left(\tau_{2}\right)+len\left(\tau_{3}\right)},$$

#### 4.5.2. Correlation Analysis

The elements of the correlation matrix are the correlation coefficients for the corresponding pairs of variables. The correlation coefficient indicates the extent to which the variables depend on each other. The session number indicates the number of hours of string operation <2 h; 30 h>.

Table 2, Table 3, Table 4, Table 5, Table 6 and Table 7 present correlation matrices and show that the most significant parameter for most strings is the time of sound decay $T_{f4}$. In addition, for string E2 (Table 2), the parameter $\sigma_{3}$ proved very significant. The correlation coefficient of the $\sigma_{3}$ parameter with the recording session number was as high as 0.82. The exception was the G string (Table 5), for which the $T_{f4}$ time is insignificant. For the G string, the parameters correlated with the recording session number were the times $\tau_{2}$ and $\tau_{3}$.

## 5. Results of the Experiment

### 5.1. Results of Experiments

**Figure 9:**
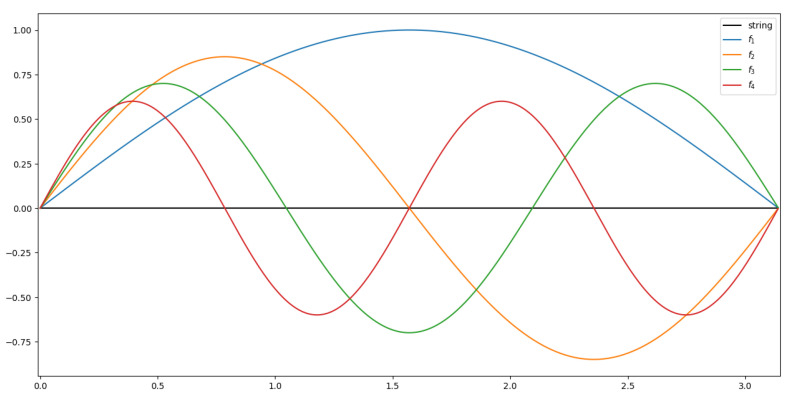
Vibration diagram of harmonic string sound.

In our opinion, the guitar strings change their linear density locally during playing. These changes may be due to dynamic tension, string pressure on the sound threshold, or modulation. The change in the thickness of the string and thus its linear mass density distribution is also confirmed by an experiment we conducted on measuring the thickness of worn strings. The change in linear mass density can be important for resonating higher harmonics. The first harmonic of a string has an oscillation period equal to the entire length of the string, as shown in Figure 9; therefore, a local change in linear density will not affect its resounding. However, a change in the linear density distribution can significantly affect the sound quality of the higher harmonics. For example, the second harmonic $f_{2}$ already has a period equal to half the length of the string. The frequency of the first harmonic may deviate from the expected value.

### 5.2. Bridge over the Volga River

The inspiration for further analysis of the phenomena occurring inside the strings of an electric guitar turned out to be a phenomenon that occurred in 2010 in a bridge on the Volga River [35], located in Volgograd. Observers recorded the phenomenon of vibration of the span and the entire bridge structure. For example, it can be viewed on YouTube—a video from 2010 [36]. What is surprising in this recording is the dynamic change in the thickness of parts of the structure. The change in the thickness of the strings in a guitar (including the change in the position of the thickened parts) seems to be similar to what was observed during the vibrations of the bridge structure. However, in the case of an electric guitar, the frequencies of the string vibrations are much higher than the frequencies of the bridge vibrations on the Volga. Those phenomena make dynamic observation of this phenomenon very difficult.

## 6. String Strength Test

### 6.1. Objective of the Experiment

This experiment evaluated the tensile strength of strings using a uniaxial tensile testing machine. The strings were subjected to static tensile testing in the machine until failure occurred. The maximum load the strings could withstand during the static test and their deformation characteristics were recorded. This procedure allowed for a thorough assessment of the strings’ mechanical properties and performance limits under controlled conditions.

### 6.2. Preparation of Strings

Five strings were used for the test. The steel strings had thicknesses of 0.25 mm, 0.33 mm, 0.43 mm, 0.66 mm, and 0.91 mm. The strings were mounted in the tensile testing machine according to [37], which defines methods for securing metal material samples in a tensile testing machine to ensure accurate and repeatable results for tensile strength tests.

**Figure 10:**
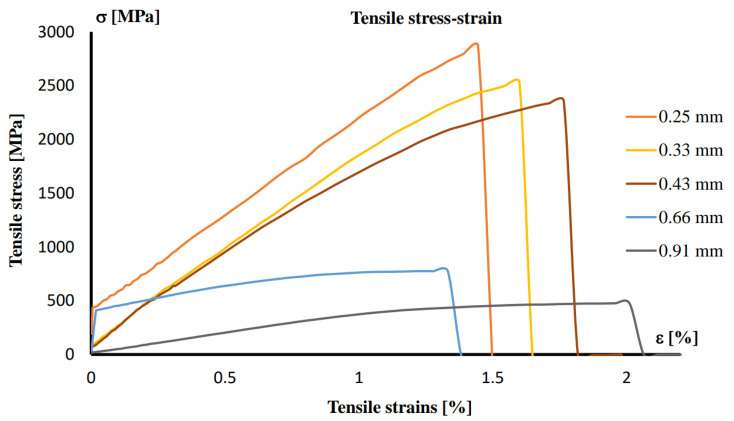
Stress–strain diagram of the strings subjected to uniaxial tensile testing.

Figure 10 presents the stress–strain diagram for the strings under uniaxial tensile testing.

### 6.3. Static Tension

We calculated the strings’ static stress to understand better the experimental results obtained. The stress [38] is expressed by the Formula (9).

(9) $$\sigma =\frac{\vec{F}}{A},$$

Knowing (10) and (11)

(10) $$A=\pi r^{2}=\pi {\left(\frac{d}{2}\right)}^{2}=\frac{\pi d^{2}}{4},$$

(11) $$\vec{F}=m\vec{a}=m\vec{g},$$

Therefore, the tension formula is of the form (12).

(12) $$\sigma =\frac{4m_{N}\vec{g}}{\pi d^{2}},$$

Knowing the formula, we calculated the stress for all the strings. Results can be found in the Table 8 and Table 9.

The stresses that occur on an electric guitar string are much greater than those we are familiar with in building structures [39,40]. For example, all commercially available wire has a yield strength of 500 MPa, while round bars are 250 MPa [41]. Our calculations and experiment show that the yield limit is exceeded immediately when the string is placed on the guitar neck. Exceeding the yield stress causes the string to deform irreversibly after each picking.

## 7. Modeling

### 7.1. String Equation

**Figure 11:**
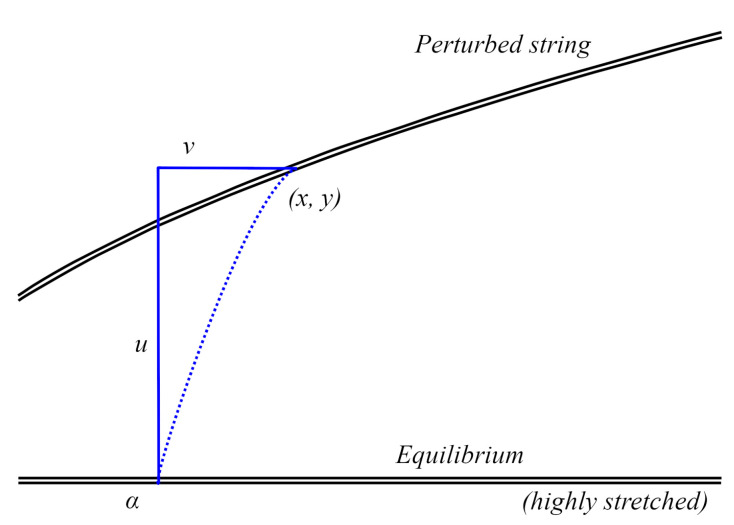
Scheme of the perturbed string.

The formula for its frequency must be introduced to understand further phenomena occurring in the string properly. As a first step, we consider a schematic depiction of the perturbed string (Figure 11).

**Figure 12:**
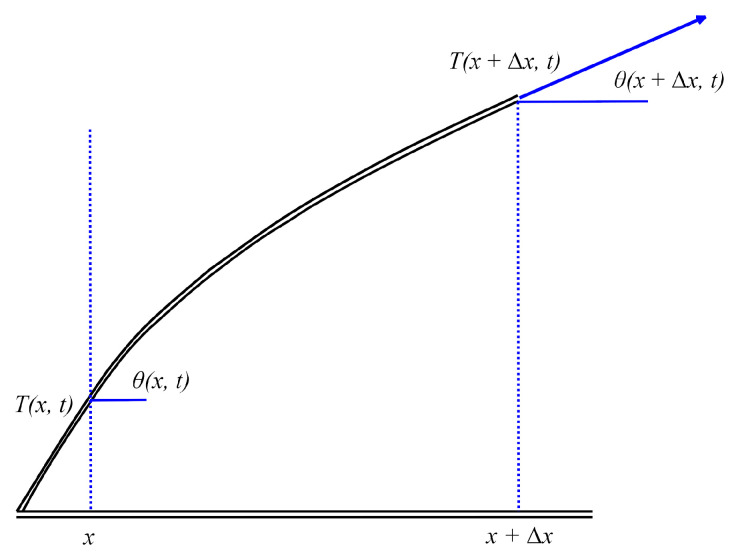
Scheme of the perturbed string assuming that the displacement is only vertical.

Simplifying that the shift is only vertical, y = u(x, t) (Figure 12).

The stress is described by Equation (13).

(13) $$\frac{dy}{dx}=\frac{\partial u}{\partial x}=tan\left(\theta \left(x,t\right)\right),$$

We know (14) from Newton’s law.

(14) $$\vec{F}=m\vec{a},$$

(15) $$\rho_{0}\left(x\right)\Delta x\frac{\partial^{2}u}{\partial t^{2}}=T\left(x+\Delta x,t\right)sin\left(\theta \left(x+\Delta x,t\right)\right)-T\left(x,t\right)sin\left(\theta \left(x,t\right)\right)+\rho_{0}\left(x\right)\Delta xQ\left(\xi ,t\right),$$

where ξ∈[x,x+Δx] and Q(ξ,t) are any “body” accelerations of the guitar, such as gravitation or resistance and $\rho_{0}\left(x\right)$ is volume density.

Dividing by Δx and assuming that Δx→0, we obtain (16).

(16) $$\rho_{0}\left(x\right)\frac{\partial^{2}u}{\partial t^{2}}=\frac{\partial }{\partial x}\left(T\left(x,t\right)sin\left(\theta \left(x,t\right)\right)\right)+\rho_{0}\left(x\right)Q\left(x,t\right),$$

For a small angle θ, we can assume (17).

(17) $$\frac{\partial u}{\partial x}=tan\left(\theta \right)=\frac{sin(\theta )}{cos(\theta )}\approx sin\left(\theta \right),$$

(18) $$\rho_{0}\left(x\right)\frac{\partial^{2}u}{\partial t^{2}}=\frac{\partial }{\partial x}\left(T\left(x,t\right)\frac{\partial u}{\partial x}\right)+\rho_{0}\left(x\right)Q\left(x,t\right),$$

Due to the elasticity of the string, we can assume (19).

(19) $$T\left(x,t\right)\approx T_{0},$$

Thus,

(20) $$\rho_{0}\left(x\right)\frac{\partial^{2}u}{\partial t^{2}}=T_{0}\frac{\partial^{2}u}{\partial x^{2}}+\rho_{0}\left(x\right)Q\left(x,t\right),$$

If Q is small,

(21) $$c^{2}=\frac{T_{0}}{\rho_{0}},$$

(22) $$\frac{\partial^{2}u}{\partial t^{2}}=c^{2}\frac{\partial^{2}u}{\partial x^{2}},$$

$$\begin{array}{cc} \mathrm{Partial}\;\mathrm{differential}\;\mathrm{equation}: & \;\frac{\partial^{2}u}{\partial t^{2}}=c^{2}\frac{\partial^{2}u}{\partial x^{2}}\\ \mathrm{Boundary}\;\mathrm{conditions}: & \;u(0,t)=0,\;u(L,t)=0\\ \mathrm{Initial}\;\mathrm{conditions}: & \;u\left(x,0\right)=f\left(x\right),\;u_{t}\left(x,0\right)=g\left(x\right) \end{array}$$

(23) $$u\left(x,t\right)=\sum_{n=1}^{\infty }\left[A_{n}cos\left(\frac{n\pi ct}{L}\right)+B_{n}sin\left(\frac{n\pi ct}{L}\right)\right]sin\left(\frac{n\pi x}{L}\right),$$

We know that

(24) $$f_{0}=\frac{c}{2L}=const,$$

(25) $$\frac{n\pi ct}{L}=\frac{2n\pi ct}{2L}=\frac{x}{2L}2n\pi t=2\pi nf_{0}t,$$

Thus, we obtain (26).

(26) $$u\left(x,t\right)=\sum_{n=1}^{\infty }\left[A_{n}cos\left(2\pi nf_{0}t\right)+B_{n}sin\left(2\pi nf_{0}t\right)\right]sin\left(\frac{n\pi x}{L}\right),$$

We can obtain the phase from this equation, described by Equation (27).

(27) $$\Theta_{n}\left(t\right)=2\pi nf_{0}t,$$

By differentiating the phase Equation (27), we obtain the formula for the frequency of the individual harmonic sound of the string (29).

(28) $$f_{n}=\frac{1}{2\pi }\cdot \frac{d\Theta_{n}}{dt}=\frac{1}{2\pi }2\pi nf_{0},$$

(29) $$f_{n}=nf_{0},$$

### 7.2. Introduction of Frequency Modulation

However, it turns out that the string’s yield limit is exceeded as soon as it is placed on the guitar post legally. Consequently, this leads us to believe that Equation (29) inadequately describes the string’s frequency. It seems necessary to introduce frequency modulation to more accurately describe the frequency of the sound produced by the string.

### 7.3. The Problem of Speed in the String

Knowing the vibration model of the string shown in Figure 9 and the fact that the string is elastic, we can see that for the first harmonic $f_{1}$ we can assume $T\left(x,t\right)=T_{0}$, since the linear density of the entire string will not change.

However, when analyzing the experimental results, we observed a change in the distribution of the linear density of the string during its exploitation. As shown in the diagram (Figure 9), the higher harmonics have a smaller period than the first harmonic. Thus, changing the linear density distribution can negatively affect the sound produced by the higher harmonics. Thus, we assume $T\left(x,t\right)=T_{0}\left(t\right)$ for higher harmonics.

Due to the elasticity of the string, we can assume (30) for $f_{1}$ and (31) for the other harmonic.

(30) $$T\left(x,t\right)\approx T_{0},$$

(31) $$T\left(x,t\right)\approx T_{0}\left(t\right),$$

Thus, we obtain (32).

(32) $$\rho_{0}\left(x\right)\frac{\partial^{2}u}{\partial t^{2}}=T_{0}\left(t\right)\frac{\partial^{2}u}{\partial x^{2}}+\rho_{0}\left(x\right)Q\left(x,t\right),$$

If *Q* is small, we can assume (33).

(33) $$c^{2}=\frac{T_{0}\left(t\right)}{\rho_{0}},$$

(34) $$\frac{\partial^{2}u}{\partial t^{2}}=c^{2}\frac{\partial^{2}u}{\partial x^{2}},$$

#### 7.3.1. Average Speed—$c_{0}$

Average velocity is defined by the following formula [42].

(35) $$v=\frac{L}{t},$$

where *v*—velocity [m/s], *L*—length [m], *t*—time [s].

In the case of pyrethroid vibration ($f_{0}$), the average sound speed covers the entire vibrating string length (from the first fret to the bridge mount). In such a situation, a local change in linear mass density does not cause a change in global linear density. A local thickening of one section of the string results in a proportional decrease in the string’s diameter elsewhere. The vibrating mass is the same all the time.

#### 7.3.2. Instantaneous Velocity—$c_{0}\left(t\right)$

For higher harmonics, a local change in density will result in a change in the speed of sound in parts of the string. The consequence of this phenomenon should be a frequency modulation visible for higher harmonics, and completely absent on the first harmonic.

To test the hypothesis of frequency modulation of higher harmonics, we modified the change in stress over time in the string equation.

### 7.4. String Equation with Modulation Introduced

Since we are observing frequency modulation, we introduce an additional function into the string equation (26) responsible for this phenomenon. Assuming it is ϕ(t), we therefore obtain (36).

(36) $$u\left(x,t\right)=\sum_{n=1}^{\infty }\left[A_{n}cos\left(2\pi nf_{0}t+\phi \left(t\right)\right)+B_{n}sin\left(2\pi nf_{0}t+\phi \left(t\right)\right)\right]sin\left(\frac{n\pi x}{L}\right),$$

From Equation (36), we can obtain the phase (37).

(37) $$\Theta_{n}\left(t\right)=2\pi nf_{0}t+\phi \left(t\right),$$

Thus, the equation for the quadratic frequency of the individual harmonics of the string will be of the form (39).

(38) $$f_{n}\left(t\right)=\frac{1}{2\pi }\cdot \frac{d\Theta_{n}}{dt},$$

(39) $$f_{n}\left(t\right)=nf_{0}+\frac{1}{2\pi }\cdot \frac{d\phi }{dt},$$

### 7.5. Modulated Signal Generation

We decided to generate a signal similar to the pitch of the sound inside time $au_{3}$. We assumed that a sinusoidal function modulates the speed of the wave. The generated signal is given by Equation (40).

(40) $$\begin{array}{cc} \; & f_{1}\left(x\right)=sin\left(2\pi f_{0}t\right)\\ \; & f_{2}\left(x\right)=sin\left(2\cdot 2\pi f_{0}t\right)+sin\left(\Omega \right)\\ \; & f_{3}\left(x\right)=sin\left(3\cdot 2\pi f_{0}t\right)+sin\left(\Omega \right)\\ \; & ....\\ \; & y=\sum_{n=1}^{\infty }f_{n}\left(x\right) \end{array}$$

Then, we applied the same pitch testing procedure to the generated signal to calculate the string’s pitch.

### 7.6. Comparison of the Pitch of the String with the Generated Signal

**Figure 13:**
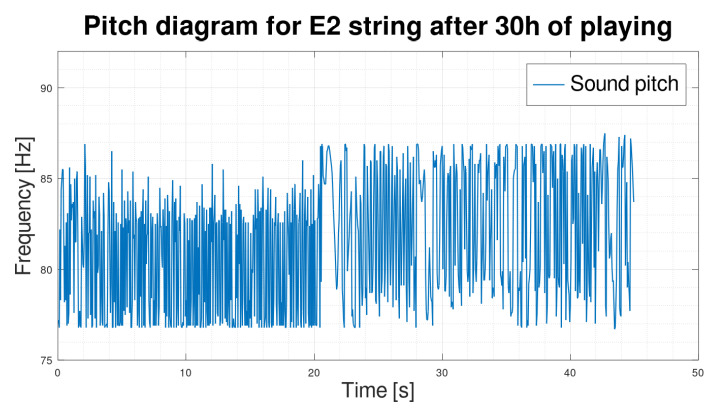
Pitch diagram of the generated string sound.

Since the aforementioned model (40) describes the behavior of the string at the moment of the end of the sounding, it was decided to conduct numerical experiments related to the last stage of the string sounding (Figure 13) and compare them to the experimental results (Figure 8).

We observed that with the adopted parameters (Ω=0.01), a modulation result was very similar (also parametrically) to the experimental results. The range of pitch changes in both cases is in the range (76 Hz–89 Hz).

The most significant difference is the appearance of frequency modulation in the recording of string vibration.

## 8. Discussion

Our research has shown that compromising string life and playing comfort is tough. Metallurgists are making every effort to achieve this compromise. At this point, extending string life while maintaining their usefulness for musicians and playing comfort will not be easy. Creating strings with extended life based on doped carbon steel seems downright impossible due to the stresses on the strings and the stresses that limit carbon steel.

However, let us focus on the strings we tested. During our research, we found that when the phenomenon described in the Section 3.5, consisting of the occurrence of significant difficulties during the tuning of the strings, caused by large pitch fluctuations, already occurs during the first seconds after the string is struck. We can say with certainty that these strings are already suitable for replacement. The musical properties of the strings have deteriorated significantly. During testing, we found that this phenomenon occurs much faster than 40 h of use. The strings should therefore be replaced faster than the 40 h of playing time described in [11]. According to this criterion, set A lasted 21 h and set B lasted 28 h of use.

In our studies of string thickness variation, we found two important phenomena. Firstly, strings without a wrapper stretch more than strings with a wrapper. However, the strings with the wrapping wear faster according to the analysis of the graphs of the pitch and the derived parameters. We found that this phenomenon is due to having a wrapper. The core of a string with a wrapper stretches presumably similarly to strings without a wrapper. However, the elongation of the string core affects the wrapper itself. This elongation causes it to loosen locally and thicken the wrapper. Thus, this loosening of the wrapping significantly affects the acoustic properties of the string.

Another important conclusion from our research concerns using a perceptual model to obtain pitch. As can be seen, the frequency of the fundamental tone appears very stable. We can only see the wear and tear when we analyze the relationships between the harmonics of the sound under study. Having previously applied the DTFT transform to the signal, this approach is a significant achievement. This approach can be applied to studying the wear and tear of similar systems, such as vibrating systems like train axles or bridges.

Further research into the phenomenon of modulation of modal frequencies as a consequence of local density variations should be carried out towards the development of a modulation envelope model, i.e., determination of parameter variations described by the Formulae (40).

## 9. Conclusions

This paper presents an innovative approach to material durability analysis using time-frequency analysis based on variations in sound pitch. The classical string equation was modified to accurately describe the changes in vibration frequency during the string’s wear process. The results demonstrate the potential of this method for broader applications, including the structural health assessment of engineering constructions such as bridges. Observing frequency modulation over time allows for more precise and significantly earlier prediction of structural failures, offering a substantial improvement over conventional modal analysis, which does not utilize time-frequency analysis tools. Based on the detailed characterization of the wear phenomenon, it is possible to develop an automatic material quality classifier, which can be implemented using neural network models. This approach opens new perspectives in the field of mechanical structure diagnostics.

## Figures and Tables

**Table 1:** Dimensions of G-string in set B.

Measurement Location	Average of Measurements [mm]	Share % Relative to the Measurement at the 12th [%]	Dimension Given by the Manufacturer [mm]	Share % Relative to Manufacturer’s Stated Dimensio [%]
12 fret	0.513	100.00%	0.508	101.05%
Behind the bridge	0.525	102.34%	0.508	103.41%
1 fret	0.511	99.55%	0.508	100.59%
By the key	0.514	100.13%	0.508	101.18%
Picking place	0.546	106.36%	0.508	107.48%

**Table 2:** Correlation matrix for the string E2 in set A.

	$\tau_{2}$	$\tau_{3}$	$T_{f4}$	$\sigma_{2}$	$\sigma_{3}$	$f_{average}$	Session Number
$\tau_{2}$	1	−1	−0.127	−0.335	0.062	−0.281	−0.093
$\tau_{3}$	−1	1	0.127	0.335	−0.062	0.281	0.093
$T_{f4}$	−0.127	0.127	1	0.026	0.034	−0.027	−0.195
$\sigma_{2}$	−0.335	0.335	0.026	1	0.177	0.264	0.185
$\sigma_{3}$	0.062	−0.062	0.034	0.177	1	0.309	0.820
$f_{average}$	−0.281	0.281	−0.027	0.264	0.309	1	0.434
Session number	−0.093	0.093	−0.195	0.185	0.820	0.434	1

**Table 3:** Correlation matrix for the string A in set A.

	$\tau_{2}$	$\tau_{3}$	$T_{f4}$	$\sigma_{2}$	$\sigma_{3}$	$f_{average}$	Session Number
$\tau_{2}$	1	−1	−0.258	−0.178	−0.232	−0.257	−0.382
$\tau_{3}$	−1	1	0.258	0.178	0.2316	0.257	0.382
$T_{f4}$	−0.258	0.258	1	0.095	0.2307	−0.165	−0.441
$\sigma_{2}$	−0.178	0.178	0.095	1	0.4198	0.008	0.119
$\sigma_{3}$	−0.232	0.232	0.231	0.420	1	−0.151	0.122
$f_{average}$	−0.257	0.257	−0.165	0.008	−0.151	1	0.325
Session number	−0.382	0.382	−0.441	0.119	0.122	0.325	1

**Table 4:** Correlation matrix for the string D in set A.

	$\tau_{2}$	$\tau_{3}$	$T_{f4}$	$\sigma_{2}$	$\sigma_{3}$	$f_{average}$	Session Number
$\tau_{2}$	1	−1	−0.124	0.084	0.429	−0.104	0.002
$\tau_{3}$	−1	1	0.124	−0.084	−0.429	0.104	−0.002
$T_{f4}$	−0.124	0.124	1	0.231	0.164	0.218	0.437
$\sigma_{2}$	0.084	−0.084	0.231	1	0.285	−0.217	0.252
$\sigma_{3}$	0.429	−0.429	0.164	0.285	1	−0.084	0.121
$f_{average}$	−0.104	0.104	0.218	−0.217	−0.084	1	0.454
Session number	0.002	−0.002	0.437	0.252	0.121	0.454	1

**Table 5:** Correlation matrix for the string G in set A.

	$\tau_{2}$	$\tau_{3}$	$T_{f4}$	$\sigma_{2}$	$\sigma_{3}$	$f_{average}$	Session Number
$\tau_{2}$	1	−1	0.353	−0.461	−0.145	−0.052	0.289
$\tau_{3}$	−1	1	−0.353	0.461	0.145	0.052	−0.289
$T_{f4}$	0.353	−0.353	1	0.134	0.297	−0.338	−0.043
$\sigma_{2}$	−0.461	0.461	0.134	1	0.492	−0.023	−0.070
$\sigma_{3}$	−0.145	0.145	0.297	0.492	1	−0.033	−0.102
$f_{average}$	−0.052	0.052	−0.338	−0.023	−0.033	1	0.057
Session number	0.289	−0.289	−0.043	−0.070	−0.102	0.057	1

**Table 6:** Correlation matrix for the string B in set A.

	$\tau_{2}$	$\tau_{3}$	$T_{f4}$	$\sigma_{2}$	$\sigma_{3}$	$f_{average}$	Session Number
$\tau_{2}$	1	−1	0.148	−0.770	0.182	0.103	−0.058
$\tau_{3}$	−1	1	−0.148	0.770	−0.182	−0.103	0.058
$T_{f4}$	0.148	−0.148	1	−0.191	0.405	0.043	0.477
$\sigma_{2}$	−0.770	0.770	−0.191	1	−0.056	−0.307	0.079
$\sigma_{3}$	0.182	−0.182	0.405	−0.056	1	0.001	0.144
$f_{average}$	0.103	−0.103	0.043	−0.307	0.001	1	0.219
Session number	−0.058	0.058	0.477	0.079	0.144	0.219	1

**Table 7:** Correlation matrix for the string E4 in set A.

	$\tau_{2}$	$\tau_{3}$	$T_{f4}$	$\sigma_{2}$	$\sigma_{3}$	$f_{average}$	Session Number
$\tau_{2}$	1	−1	−0.277	−0.396	−0.171	0.342	0.373
$\tau_{3}$	−1	1	0.277	0.396	0.171	−0.342	−0.373
$T_{f4}$	−0.277	0.277	1	0.045	0.406	−0.298	−0.372
$\sigma_{2}$	−0.396	0.396	0.045	1	−0.018	0.112	−0.002
$\sigma_{3}$	−0.171	0.171	0.406	−0.018	1	−0.103	−0.199
$f_{average}$	0.342	−0.342	−0.298	0.112	−0.103	1	0.182
Session number	0.373	−0.373	−0.372	−0.002	−0.199	0.182	1

**Table 8:** Calculated stress table in set A.

*String*	$m_{N}\;$[kg]	d[m]	*Stress* [MPa]
E2	7.67	0.0011684	70.18
*A*	8.67	0.0009144	129.07
*D*	8.34	0.0006604	238.85
*G*	7.52	0.0004318	503.77
*B*	6.98	0.0003302	799.61
E4	7.36	0.0002540	1424.92

**Table 9:** Calculated stress table in set B.

*String*	$m_{N}\;$[kg]	d[m]	*Stress* [MPa]
E2	9.24	0.0012192	77.64
*A*	10.15	0.0009652	136.09
*D*	9.92	0.0007112	244.97
*G*	9.4	0.0005080	454.97
*B*	8.09	0.0003556	799.11
E4	7.36	0.0002540	1424.92

**Figure A1:**
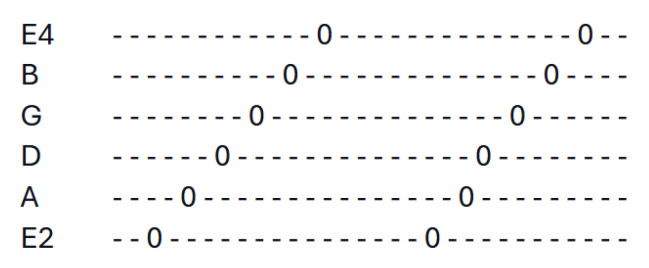
Tablature showing the recording session’s scheme.

**Figure A2:**
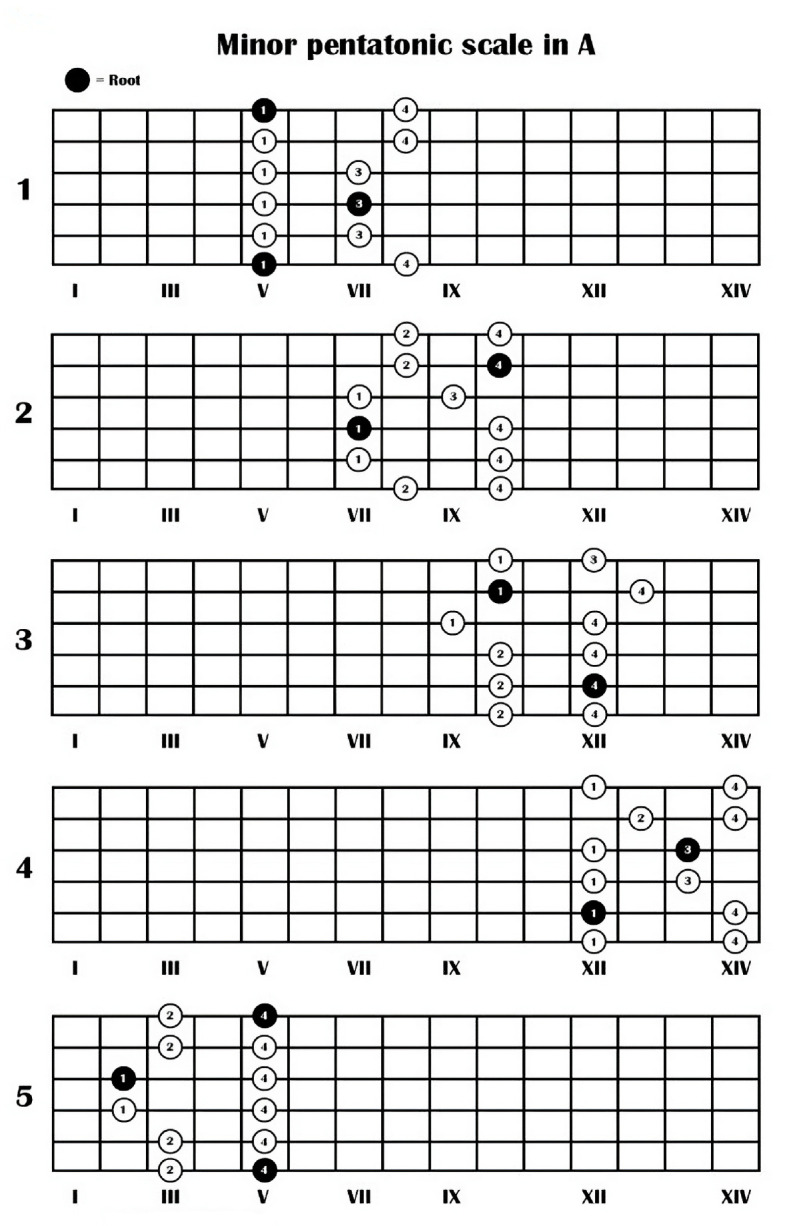
Pentatonic scales.

**Figure A3:**
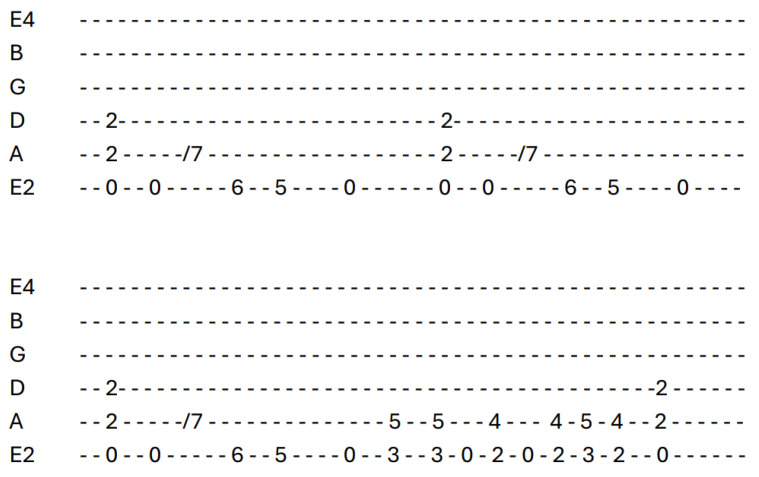
First guitar riff.

**Figure A4:**
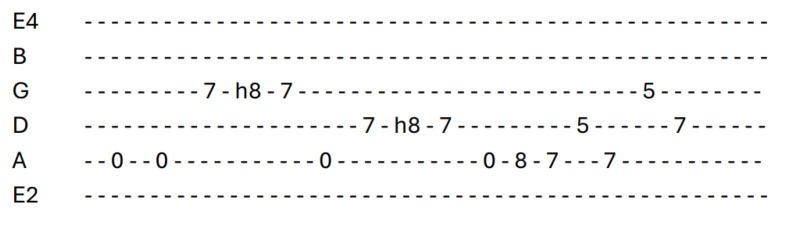
Second guitar riff.

**Figure A5:**
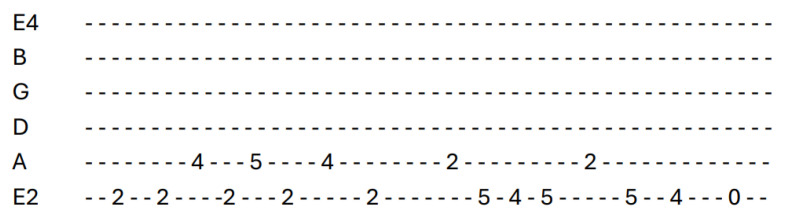
Third guitar riff.

**Figure A6:**
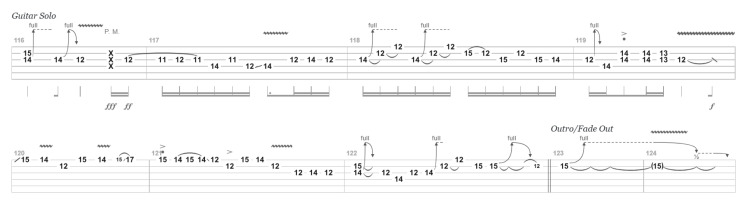
First guitar solo.

**Figure A7:**
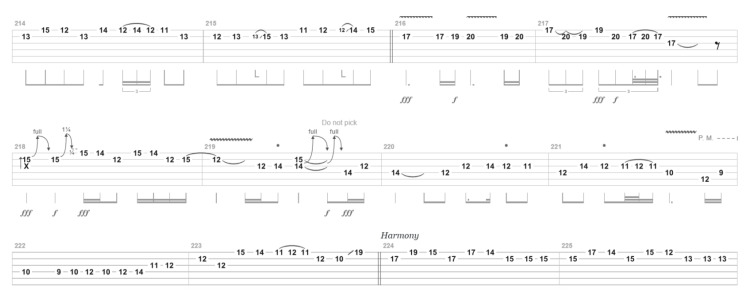
Second guitar solo.

## Data Availability

All data used in this paper, including recordings, are available from the authors upon request.

## Appendix A. Recording Session Detailed Description

### Appendix A.1. What Did the Single Recording Session Look Like?

A single recording session consisted of recording the sound of each string. The sound of an empty string was recorded. That is, the sound of a string not pressed against the fret, the so-called ’0’ fret. The sound of each string was recorded until it ceased to sound. For the E2–D strings, this was about 40–60s of recording. For the G–E4 strings, it was about 30–20s of recording. Each string was tuned correctly before the recording session. The string was hit by the musician with more or less the same force. In order to compensate for the difference in striking force or other unwanted environmental changes affecting the sound of the string, each sound was recorded 2 times.

The diagram of the recording session can be illustrated using the tablature (Figure A1).

### Appendix A.2. Guitar Playing Session

According to what we wrote in the article, the single session of guitar playing lasted one hour. To use the strings more evenly, we decided to divide the playing hour into three twenty-minute sections. During these sections, we played pentatonics, riffs, and solos, respectively. This methodology aims to use each string as evenly as possible. At the same time, the way of playing is usable all the time. If we had not divided the playing session into these three sections, one of the strings would have been used far more than the others. Using one of the strings more heavily could have caused an erroneous picture of string wear.

Using one of the strings more heavily could reduce the usefulness of the information in the paper for musicians. For example, in metal music, the E2 string is used by far the most. Additionally, aggressive picking can cause excessive string wear. However, in jazz music, all strings are used evenly, and the same applies to pop music. Therefore, it was necessary to normalize the session so that all strings were used evenly.

We will now describe each section in detail.

#### Appendix A.2.1. Pentatonics Scales

A pentatonic scale is a scale made up of 5 notes within an octave. The central sound of a pentatonic scale is the middle sound, supplemented by intervals of a major second and a diminished fourth, both up and down. The pentatonic scales engaged all the strings of the guitar equally. One of the pentatonic scales is presented in Figure A2 form site [43].

#### Appendix A.2.2. Guitar Riffs

A riff in music refers to a short melody, motif, or single phrase repeated many times within a song. Most guitar riffs are played on the E2, A, and D strings.

We will now show some examples of guitar riffs that we played during this section (Figure A3, Figure A4 and Figure A5).

As we can see in Figure A3, Figure A4 and Figure A5, during the twenty-minute section, the guitar riffs mainly used the E2, A, and D strings.

#### Appendix A.2.3. Guitar Solos

A guitar solo is a piece of music played by a single artist. To distinguish this part of the song from the rest, higher notes are often used than in the rest of the song.

We will now show a sample tablature of guitar solos played by us while experimenting.

As we can see in Figure A6 and Figure A7, the guitar solos mainly engaged the G, B, and E4 strings.

Dividing an hour-long playing session into twenty-minute sessions allowed us to use the strings more evenly. At the same time, since we were playing parts of existing songs, the way of playing remained as similar as possible to the “real” one.
